# Critical Domains Within the Self-Reported Patient Experience of Virtual Care

**DOI:** 10.1001/jamanetworkopen.2023.54159

**Published:** 2024-01-31

**Authors:** Kori S. Zachrison, Zhiyu Yan, Yizhou Cui, Lee Park, Lee H. Schwamm

**Affiliations:** 1 Department of Emergency Medicine, Massachusetts General Hospital, Boston; 2 Department of Biostatistics, Harvard T.H. Chan School of Public Health, Boston, Massachusetts; 3 Department of Internal Medicine, Brigham & Women’s Hospital, Boston, Massachusetts; 4 Mass General Brigham, Boston, Massachusetts; 5 Department of Neurology, Yale School of Medicine, New Haven, Connecticut

## Abstract

This cross-sectional study examines the association between domains of patient experience with a physician and patient likelihood of recommending the physician for virtual vs in-person visits.

## Introduction

There is evidence that patients are generally satisfied with virtual care.^1,2,3^ However, little is known about logistical or interpersonal components of the patient experience that are associated with the commonly used summary measure of “likelihood to recommend.” Our primary objective was to characterize whether patient-reported experience varied between virtual and in-person visits with respect to 3 domains of experience: (1) clinician listening carefully, (2) clinician explaining things clearly, and (3) clinician treating patient with courtesy and respect. We then examined the association between these domains of patient experience with overall patient experience rating after accounting for visit, patient, and physician characteristics.

## Methods

This cross-sectional study was a retrospective analysis of administrative data from Mass General Brigham, a large regional health care system, including all ambulatory visits from October 1, 2020, to September 30, 2021, with patient experience scores recorded. The study was approved by the Mass General Brigham institutional review board, and informed was waived for this retrospective use of data. We followed the STROBE reporting guideline. We compared experiences between virtual (video or audio-only) and in-person encounters for each experience domain and their contribution to overall likelihood of recommending the physician to friends or family. We used generalized linear mixed models to characterize the association between visit modality and physician and patient characteristics that were associated with each of the 3 experience domains, accounting for clustering by physician. The final model examined the contribution of these domains to the overall likelihood to recommend the physician, after accounting for physician and patient characteristics, ease of scheduling, and clustering by physician. Analyses were conducted in RStudio version 4.1.2 (R Project for Statistical Computing), and hypothesis testing was 2-sided and *P* < .05 indicated statistical significance. Additional methodological details appear in the eAppendix in Supplement 1.

## Results

Of 417 828 included encounters with 3462 physicians during the study period, 327 158 (78%) were in-person, 74 926 (18%) video, and 14 744 (4%) audio-only; 60% of encounters were among female patients, and the mean (SD) patient age was 62.3 (18.0) years. Encounters with patient experience data were less often audio-only and were made by patients who were slightly older and less often Hispanic, preferring another language, or with Medicaid insurance. Patient race, ethnicity, language preference, and portal activation also varied by encounter type (Table 1). Overall, 378 824 encounters (91%) received a top-box score for overall likelihood to recommend the physician. Patient experience varied between in-person, video, and audio-only visits and was notably lower for audio-only on all domains measured. When modeling all 3 domains (listened carefully, explained things, and treated with courtesy and respect), both video and audio-only visits had lower odds of a “yes, definitely” response after adjustment for patient and physician characteristics. Factors such as belonging to a minoritized racial or ethnic group, preferring a language other than English, having Medicaid for insurance, and lack of patient portal activation were all associated with lower odds of a “yes, definitely” response (Table 2). When examining factors associated with overall likelihood to recommend a physician, the strongest association was with communication skills (Table 2). Audio-only visits had lower likelihood to recommend the physician overall (unadjusted odds ratio [OR], 0.82; 95% CI, 0.77-0.87); however, this was entirely explained by patient experience domains (adjusted OR including patient experience, 1.67; 95% CI, 1.25-2.23). The association remained similar with addition of patient and physician characteristics to the model (fully adjusted OR, 1.57; 95% CI, 1.14-2.17) (Table 2).

**Table 1.** Characteristics of Encounters Overall and by Visit Modality

Characteristic	Individuals, No. (%)				*P* value
	Overall (N = 417 828)	In-person (n = 327 158)	Virtual video (n = 75 926)	Audio-only (n = 14 744)	
Patient					
Age, mean (SD), y	62.3 (18.0)	62.9 (17.9)	58.8 (18.4)	66.6 (15.5)	<.001
Gender					
Woman	249 614 (59.7)	194 329 (59.4)	46 420 (61.1)	8865 (60.1)	<.001
Man	168 214 (40.3)	132 829 (40.6)	29 506 (38.9)	5879 (39.9)	
Race					
Asian	13 504 (3.4)	10 420 (3.3)	2812 (3.8)	272 (2.0)	<.001
Black	18 551 (4.6)	14 626 (4.7)	3166 (4.3)	759 (5.5)	
White	352 008 (87.9)	274 854 (87.8)	65134 (88.4)	12 020 (86.9)	
Other^a^	16 330 (4.1)	13 010 (4.2)	2540 (3.4)	780 (5.6)	
Hispanic ethnicity	21 067 (5.5)	16 663 (5.6)	3138 (4.5)	1266 (9.3)	<.001
Prefers language other than English	20 356 (4.9)	17 096 (5.2)	2050 (2.7)	1210 (8.2)	<.001
With Medicaid	22 242 (5.4)	17 312 (5.4)	3782 (5.0)	1148 (7.8)	<.001
Patient portal not activated	62 979 (15.2)	57 372 (17.7)	1219 (1.6)	4388 (30.1)	<.001
Physician					
Gender					
Woman	183 106 (44.3)	142 427 (44.1)	35 313 (46.8)	5366 (36.6)	<.001
Man	234 722 (55.7)	184 731 (55.9)	40 613 (53.2)	9378 (63.4)	
Major teaching hospital affiliation	285 081 (68.2)	211 154 (64.5)	64 678 (85.2)	9249 (62.7)	<.001
Specialty					
Primary care	114 643 (27.5)	95 127 (29.2)	15 591 (20.5)	3925 (26.6)	<.001
Medical	178 593 (42.9)	129 965 (39.9)	42 338 (55.8)	6290 (42.7)	
Surgical	117 095 (28.1)	100 707 (30.9)	13 210 (17.4)	3178 (21.6)	
Behavioral health	6407 (1.5)	291 (0.1)	4776 (6.3)	1340 (9.1)	
Proportion of patients self-pay/Medicaid, mean (SD)	9.0 (8.6)	8.9 (8.8)	9.2 (7.5)	10.6 (10.6)	<.001
Proportion of patients non-English preferring, mean (SD)	6.6 (7.0)	6.7 (7.0)	5.8 (5.2)	8.2 (11.7)	<.001
Proportion of patients with activated portal, mean (SD)	82.0 (12.6)	80.4 (12.9)	89.1 (7.3)	79.5 (12.8)	<.001

^a^ Other race includes race documented as other and race not documented.

**Table 2.** Factors Associated With Patient Experience Ratings in Adjusted Analyses

Characteristic	Listened carefully, OR (95% CI)	Explained things understandably, OR (95% CI)	Treated with courtesy and respect, OR (95% CI)	Likelihood to recommend physician, OR (95% CI)
Visit modality (reference group, in-person)				
Video	0.78 (0.75-0.80)	0.84 (0.81-0.86)	0.80 (0.77-0.83)	1.06 (0.93-1.20)
Phone	0.54 (0.51-0.56)	0.58 (0.55-0.61)	0.56 (0.53-0.60)	1.57 (1.14-2.17)
Physician characteristics				
Age, per 1-year increase	0.98 (0.97-0.99)	0.98 (0.97-0.99)	0.98 (0.96-1.00)	1.08 (1.03-1.14)
Woman	1.04 (1.00-1.09)	1.07 (1.02-1.11)	1.04 (0.96-1.11)	1.05 (0.95-1.15)
Major teaching hospital affiliation	0.98 (0.95-1.00)	1.05 (0.99-1.11)	1.05 (1.00-1.11)	1.08 (0.73-1.58)
Specialty (reference group, primary care)				
Medical	0.97 (0.92-1.03)	0.89 (0.84-0.94)	1.00 (0.94-1.06)	1.10 (0.95-1.28)
Surgical	0.91 (0.86-0.96)	0.84 (0.79-0.89)	0.91 (0.85-0.97)	0.65 (0.47-0.91)
Behavioral health	0.79 (0.70-0.89)	0.73 (0.65-0.82)	0.82 (0.72-0.93)	0.68 (0.51-0.91)
No. of unique patients during study year (per additional patient)	0.99 (0.98-0.99)	0.99 (0.98-0.99)	0.99 (0.98-0.99)	0.96 (0.95-0.97)
Proportion of patients self-pay or Medicaid (per additional 1%)	0.94 (0.91-0.97)	0.93 (0.89-0.96)	0.95 (0.91-1.00)	1.03 (0.92-1.15)
Proportion of patients over 65 (per additional 1%)	1.00 (0.99-1.01)	1.01 (0.99-1.03)	1.01 (0.99-1.03)	0.98 (0.94-1.02)
Proportion of patients non-English preferring (per additional 1%)	0.97 (0.93-1.01)	0.96 (0.93-1.00)	0.96 (0.91-1.00)	0.93 (0.83-1.06)
Proportion of patients with activated portal (per additional 1%)	1.03 (1.01-1.05)	1.04 (1.02-1.06)	1.03 (1.00-1.06)	0.91 (0.83-1.01)
Patient characteristics				
Age (per 1-year increase)	0.95 (0.95-0.96)	0.92 (0.91-0.93)	0.93 (0.92-0.94)	1.19 (1.16-1.23)
Female	0.81 (0.79-0.82)	0.84 (0.82-0.85)	0.83 (0.80-0.86)	0.93 (0.84-1.02)
Race (reference group, White)				
Asian	0.61 (0.58-0.64)	0.55 (0.52-0.58)	0.53 (0.50-0.57)	0.65 (0.54-0.79)
Black or African American	0.60 (0.58-0.63)	0.58 (0.55-0.60)	0.50 (0.47-0.53)	1.02 (0.84-1.24)
Other	0.76 (0.71-0.80)	0.74 (0.70-0.79)	0.70 (0.65-0.75)	0.97 (0.76-1.24)
Hispanic ethnicity (reference group, non-Hispanic)	0.97 (0.91-1.03)	0.96 (0.90-1.02)	0.98 (0.91-1.06)	1.60 (1.26-2.04)
Preferred language not English (reference group, English preferring)	0.65 (0.61-0.68)	0.61 (0.58-0.64)	0.60 (0.56-0.64)	1.09 (0.86-1.37)
Medicaid or self-pay insurance	0.80 (0.77-0.84)	0.79 (0.75-0.83)	0.76 (0.72-0.81)	1.13 (0.93-1.36)
Patient portal not activated (reference group, activated)	0.42 (0.41-0.43)	0.42 (0.41-0.43)	0.35 (0.34-0.36)	1.07 (0.92-1.24)
Domains of patient experience				
Ease of scheduling	NA	NA	NA	2.14 (1.92-2.37)
Clinician listened carefully	NA	NA	NA	5.41 (4.65-6.31)
Clinician explained things clearly	NA	NA	NA	2.49 (2.12-2.93)
Clinician treated with respect	NA	NA	NA	1.03 (0.88-1.20)

Abbreviations: NA, not applicable; OR, odds ratio.

## Discussion

We found that 2 components of the patient experience had the strongest association with patients’ overall indication of their likelihood to recommend a physician: when patients reported that clinicians listen carefully and that they explain things well. While patient experience ratings were similar for in-person and video visits, they were lowest for audio-only visits, likely due to challenges with effective patient-physician communication during these encounters. Data were from a single academic integrated delivery network and may not be generalizable to other settings. In addition, our health system only sends surveys and collects data from successfully completed visits; thus, our results may be impacted by selection bias if visits that failed to occur at all due to technical issues were not included. Given the well-described disparities in patients selected for or choosing audio-only visits,^3,4,5,6^ health systems should consider training in effective communication strategies for providers when audio-only visits are necessary to improve audio-only patient experience and reduce health disparities.

## Notes

Supplement 1. **eAppendix. **Supplementary Methods
eReferences.

Supplement 2.
Data Sharing Statement
