# Supplementary material for: Critical Domains Within the Self-Reported Patient Experience of Virtual Care
Source: JAMA Netw Open. 2024 Jan 31;7(1):e2354159. doi: 10.1001/jamanetworkopen.2023.54159 (PMC10831569; doi:10.1001/jamanetworkopen.2023.54159)
Supplement: Supplement 1. — eAppendix. Supplementary Methods eReferences. [file jamanetwopen-e2354159-s001.pdf]

Supplemental Online Content

Zachrison KS, Yan Z, Cui Y, Park L, Schwamm LH. Critical domains within the self-reported patient experience of virtual care. *JAMA Netw Open.* 2024;7(1):e2354159. doi:10.1001/jamanetworkopen.2023.54159

eAppendix. Supplementary Methods

eReferences.

This supplemental material has been provided by the authors to give readers additional information about their work.

© 2024 Zachrison KS et al. *JAMA Network Open.*

eAppendix. Supplementary Methods

Setting, Population and Sources of Data

We used data from Mass General Brigham, a large regional health care system including 454 outpatient practice sites affiliated with 12 academic and community hospitals. The study was approved by our organizational IRB (protocol #2021P001413) and follows the STROBE reporting guideline for observational studies. We used enterprise-wide electronic health record data (Epic Systems), system-wide data on patient experience during ambulatory visits (NRC Health), and data from our organizational master provider credentialing database to extract relevant physician characteristics.

We used the electronic health record data to identify all ambulatory patient encounters with physicians during the one-year study period from 10/1/2020-09/30/2021. Visits were categorized as virtual (including video or audio-only) versus in-person. Visits were scheduled as in-person or virtual visits, and ICD-10 code telehealth billing modifiers were added by providers to attest to video or audio-only modality. NRC Health reaches out to survey all patients who have completed outpatient encounters via email or called on the telephone 3 days after their visit. The initial survey is sent via email, and if there is no response within 24 hours or if the patient has not provided an email address, then a phone call is made. Up to two outreaches are made for the majority of sites. The survey methodology includes exclusions to avoid over-sampling and survey fatigue; for example if a patient sees a physician in clinic and has a follow-up visit two weeks later, they would not get another survey. Similarly if a patient has multiple visits within a short period of time (e.g., seeing primary care, cardiology and rheumatology specialists on the same day) they would only receive one survey. Surveys are conducted in patients' preferred language for the seven most common languages of our patient population (English, Spanish, Portuguese, Arabic, Haitian-Creole, Russian and traditional Chinese); all other patients receive the English survey. We included all visits that had patient experience data with a recorded response for "Would you recommend this physician to your friends and family?"

Outcomes of interest

Outcomes of interest were at the visit level, based on patient responses to patient experience questions. The survey includes a number of questions about the patient's experience with the provider, the team and the facility. The questions that were included in this analysis were: (1) "How likely would you be to recommend this provider to your family and friends?" (2) "Did this care provider listen carefully to you?" (3) "Did this care provider explain things in a way you could understand?" (4) "Were you treated with courtesy and respect by this care provider?"

Response options are defined by the vendor for all health systems and are based on a 4-point ordinal scale: (1) No; (2) Yes, somewhat; (3) Yes, mostly; (4) Yes, definitely. We examined these questions with a dichotomous outcome, comparing "Yes, definitely" responses versus all other responses. We took a

hierarchical approach to evaluating patient experience, using likelihood to recommend as the final global outcome of interest as has been previously used by many industries and in healthcare.[1–3]

Other variables of interest

Other variables of interest included encounter-level variables of patient age, gender, race, ethnicity, language preference, insurance status, and presence of an active account in our electronic health patient portal (Epic MyChart). Encounters were also categorized as primary care, medical specialty, surgical specialty or behavioral health visits using the primary specialty of the physician performing the visit.

Physician-level variables of interest were age and popularized generational demographic cohort (Silent Generation: 1928-1945; Baby Boomers: 1946-1964; Generation X: 1965-1980; Millennials: 1981-1996) using the Brookings Institute classification.[4] We also characterized physicians by gender, years since medical school graduation, and academic practice (present if primary affiliation with one of our major teaching hospitals). We created a set of physician level attributes derived from characteristics of the patients they treated during the study period. This included the number of unique patients with whom they had any type of visit during the study period, and the proportion of patients with the following attributes that reflect potential social determinants of health for in-person or virtual care: self-pay or Medicaid insurance, aged 65 years or older, prefer speaking a language other than English, self-report belonging to a racial or ethnic minority group, and have an active electronic patient portal account.

Statistical Analysis

We used standard descriptive statistics to characterize the distribution of responses to patient experience questions, stratified by encounter type. We used descriptive and bivariate statistics to characterize the sample of visits overall and to examine differences in encounter characteristics between those with a top-box “yes, definitely” response versus all other responses.

To understand the relationship between visit type and each of the patient experience domains of interest after accounting for other characteristics of patients and physicians, we used generalized linear mixed models including random effect for physician to account for clustering of scores within physician. Variables included in the models were chosen a priori and were: visit modality, patient age, gender, race, ethnicity, language preference, insurance (dichotomized as self-pay or Medicaid vs. other), portal activation, and the following physician characteristics: age, gender, major teaching hospital affiliation, specialty class, number of unique patients care for during the study period, and proportion of patients self-pay or on Medicaid, over 65 years, non-English speaking, and with an activated patient portal. A separate model was examined for each of the 3 patient experience domains of interest.

Next, we were interested in understanding the relationship between these 3 patient experience domains of interest and the summary global measure of patient experience (likelihood to recommend). We used a generalized linear mixed model, with a random effect for physician to account for clustering of scores within physician. In this model, the outcome of interest was overall likelihood to recommend. Variables of interest included in the model were chosen a priori. Encounter, patient and physician variables of interest were: visit modality, patient age, gender, race, ethnicity, language preference, insurance (dichotomized as self-pay or Medicaid vs. other), portal activation, and the following physician characteristics: age, gender, major teaching hospital affiliation, specialty class, number of unique patients care for during the study period, and proportion of patients self-pay or on Medicaid, over 65 years, non-English speaking, and with an activated patient portal. A separate model was examined for each of the 3 patient experience domains of interest. We additionally included the 3 patient experience domains of interest (provider listened carefully, provider explained things clearly, and provider treated with respect) as well as an additional patient experience question from the survey measuring ease of scheduling.

All analyses were conducted in RStudio (Posit, PBC) and hypothesis testing was two-sided.

© 2024 Zachrison KS et al. *JAMA Network Open.*

eReferences

1. The One Number You Need to Grow. Accessed December 6, 2023. https://hbr.org/2003/12/the-one-number-you-need-to-grow

2. Rademakers J, Delnoij D, De Boer D. Structure, process or outcome: which contributes most to patients' overall assessment of healthcare quality? *BMJ Qual Saf*. 2011;20(4):326-331. doi:10.1136/BMJQS.2010.042358

3. Holzman SA, Davis-Dao CA, Khoury AE, Fortier MA, N Kain Z. Telemedicine and patient satisfaction during the COVID-19 pandemic: A case-control study of outpatient pediatric urology patients. *J Child Health Care*. Published online 2021. doi:10.1177/13674935211058272

4. Metropolitan Policy Program. Accessed May 23, 2021. https://www.brookings.edu/program/metropolitan-policy-program/

© 2024 Zachrison KS et al. *JAMA Network Open.*
