# Supplementary material for: Critical Domains Within the Self-Reported Patient Experience of Virtual Care
Source: JAMA Netw Open. 2024 Jan 31;7(1):e2354159. doi: 10.1001/jamanetworkopen.2023.54159 (PMC10831569; doi:10.1001/jamanetworkopen.2023.54159)
Supplement: Supplement 2. — Data Sharing Statement [file jamanetwopen-e2354159-s002.pdf]

Data Sharing Statement

Zachrison. Critical Domains Within the Self-Reported Patient Experience of Virtual Care. *JAMA Netw Open*. Published January 31, 2024. doi:10.1001/jamanetworkopen.2023.54159

Data
Data available: No

Additional Information
Explanation for why data not available: Includes PHI
